# Systematic Review and Meta: Analysis of Aortic Graft Infections following Abdominal Aortic Aneurysm Repair

**DOI:** 10.1155/2020/9574734

**Published:** 2020-01-31

**Authors:** O. S. Niaz, A. Rao, D. Carey, J. R. Refson, A. Abidia, P. Somaiya

**Affiliations:** Department of Vascular Surgery, Princess Alexandra Hospital, Harlow CM20 1QX, UK

## Abstract

*Introduction*. Aortic graft infection (AGI) is a rare complication following AAA repair and is associated with high morbidity and mortality. Management is variable, and there are no evidence-based guidelines. The aim of this study was to systematically review and analyse management options for AGI.

**Methods:**

Data was collected between July and August 2018. A full HDAS search was conducted on the following databases: MEDLINE, EMBASE, CINAHL, and PUBMED. Meta-analysis was conducted using RevMan 5 software.

**Results:**

1,365 patient outcomes were assessed (10 cohort studies and 12 comparative studies). The most common treatment was in situ replacement of the graft (ISR) followed by extra-anatomical replacement (EAR). Various grafts were used for ISR, such as fresh/cryopreserved allograft, venous graft, and prosthetic grafts. No graft material was shown to be superior. Axillobifemoral graft was the commonest type of EAR used. In the majority of cohort studies, ISR was the main treatment for AGI. There was no significant difference in the overall mortality rate (ISR *n* = 70/176 vs. EAR *n* = 70/176 vs. EAR *P* = 0.87). Graft occlusion rate was significantly lower in the ISR group vs. the EAR group (*n* = 70/176 vs. EAR *n* = 70/176 vs. EAR *P* = 0.87). Graft occlusion rate was significantly lower in the ISR group vs. the EAR group (*n* = 70/176 vs. EAR *n* = 70/176 vs. EAR *P* = 0.87). Graft occlusion rate was significantly lower in the ISR group vs. the EAR group (*Discussion*. In situ replacement is the preferred method of treatment as it had lower rates of occlusion. Further strong evidence is required, such as a multicentre trial to establish a management pathway for the condition.

## 1. Introduction

Abdominal aortic aneurysms are a focal dilatation of the aorta (dilatation at least one and a half times the width of the aorta) to a diameter greater than 3 cm. Current guidance recommends surgery on AAA greater than 5.5 cm. Until this point, the risks of surgery outweigh benefits [1]. Elective aortic aneurysm surgery has become more frequent over the last few decades due to the greater number of aneurysms being detected incidentally and via screening [2]. In 2016, 4153 elective AAA procedures were carried out across the UK [3].

Aortic graft infection (AGIs) is the infection of the primary prosthesis. This can include a graft used in open surgery or an endovascular stent used in EVARs. Seeger described the major and minor criteria to diagnose aortic graft infections [4].

There is no agreed consensus on the best management option [5]. The “gold standard” is to surgically remove the infected graft [6]; however, whether this is not possible treating with medical management is acceptable. The surgeon needs to be aware that the aortic tissue may be friable to clamp due to sepsis or atherosclerosis when performing surgery [7].

The following two main surgical methods are used:

[1] *Extra Anatomical Replacement (EAR)*. This is the revascularisation of the lower limbs by creating an extra-anatomic connection from a proximal to a distal artery—usually axillary to femoral artery. EAR is used to treat graft infections for patients with previous abdominal surgery and scarring or those at a high risk of aortic cross clamping/unsuitable for long operations such as patients with significant co-morbidities.

[2] *In Situ Replacement*. This accepted current gold standard operation is the removal of the source of infection and replacement of the infected graft with conduit grafts. These may be biological or prosthetic:

(a) Biological grafts can be split into the following:
  - Autologous: FV, GSV, and arm veins
  - Nonautologous: homografts, xenografts, and allografts
(b) Prosthetic grafts include Dacron, PTFE, and polyurethane [8]

The common clinical practice is the use of in situ replacement, whereas extra-anatomical replacement is less frequently performed [9] [10]. In the nonautologous allografts, either fresh or cryopreserved allografts were used. Fresh allografts were specimens used from fresh cadavers without preservation.

There is no Level 1 evidence to guide the choice of intervention. There is no agreed evidence-based consensus on the choice of the graft material used although it is believed that biological grafts are better as a conduit.

Associated mortality is high, with recent studies reporting it being up to 28% at 1 year [11]. Risks of surgery include local and systemic complications. Local complications include graft rupture/leak whereas systemic complications include limb loss, renal failure, and stroke.

## 2. Materials and Methods

Data was collected between July and August 2018. A full HDAS search was conducted on the following databases; MEDLINE, EMBASE, CINAHL, and PUBMED.

A PICO (Patient/problem; Intervention/exposure; Comparison; Outcome) search strategy was discussed, and agreement was reached to use broad terms. The search strategies for each database are detailed in Table 1.

**Figure 1:**
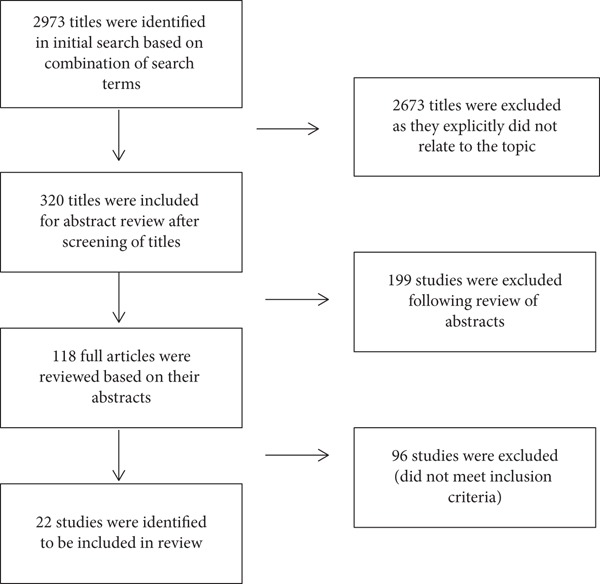
PRISMA diagram for the selection of studies included in the review.

Our initial search yielded 2973 studies. 118 full-text papers were assessed for eligibility, out of which 96 were excluded (Figure 1). Study selection and data extraction were undertaken by two investigators.

The remaining 22 articles were included and analysed by looking for the inclusion of the following research interests:

(1) Type of complications occurring postoperatively:
  - Localised complications (graft infection, rupture, or leak)
  - Systemic complications (renal failure, myocardial infarction, septic shock, and limb amputation)
(2) Intervention used to treat the following:
  - ISR
  - EAR
  - Medical management
(3) Mortality outcomes
(4) Morbidity outcomes:
  - Local complications: infection of graft post operatively
  - Local complications: wound complication
  - Local complications: graft-related complication such as rupture/leak
  - Systemic complications: myocardial Infarction
  - Systemic complications: renal failure
  - Systemic complications: stroke
  - Systemic complications: limb salvage required
(5) Duration of stay in hospital (as a marker for severity of complication)

The inclusion criteria were deliberately broad in order to allow for maximum results:

1. Studies including patients over the age of 18 years who previously had open repair or endovascular aortic repair with any form of complication encountered
2. Studies with a particular focus on morbidity and/or mortality outcomes
3. Studies that focused on the specific complications, for example, localised graft infections or systemic myocardial infarctions

The exclusion criteria were as follows:

1. Studies that did not include information as to patient outcome (morbidity or mortality statistics)
2. Case studies or case series, not deemed to be gold standard research options, therefore less valuable information

### 2.1. Demographics

1,365 patients were analysed; in this group, age ranged from 57 to 71 years, and gender was largely male. Ethnicity was not considered for the sample group, nor was occupation as they were not viewed as relevant to patient outcomes. All studies were Cochrane approved, published in reputable journals, and conducted in tertiary vascular centres across Europe, America, and Asia.

## 3. Results

1,365 patient outcomes were assessed; there were 10 cohort studies and 12 comparative studies. The most common treatment was in situ replacement of the graft (ISR) followed by extra-anatomical replacement (EAR) and conservative management. Axillobifemoral graft was the commonest type of EAR utilised. Conservative management consisted of intravenous antibiotics with or without the use of radiological drainage. The follow-up period was variable, ranging from 0 to 7 years. Twenty were conducted in a single centre. The most common outcome measure was overall mortality, followed by amputation and graft-related complications. Most did not mention whether the initial operation was an open or endovascular repair or whether operations were planned electively or done as an emergency. The common bacterial organisms identified with graft infection were gram-positive cocci, gram-negative cocci, and polymicrobials; these are summarised in Table 2 [6] [12] [13] [14] [15] [16].

There were 12 comparative studies (*n* = 608), and most of them are compared with in situ replacement of the graft with other treatments (Table 3). Two studies were multicentre [13] [17]; the rest were single-centred. Most were compared with in situ replacement (ISR) with extra-anatomical replacement (EAR) [17] [18] [19] [20]. A comparison was also made between surgical management and medical treatment with intravenous antibiotics, with or without drainage of purulent collections [21] [22] [23]. A further comparison was also made between different types of graft used for ISR; biological grafts such as an allograft (fresh or cryopreserved) were compared to prosthetic graft (including rifampicin soaked and silver coated). No study detailed the use of autologous vein as a conduit.

There were 10 cohort studies (*n* = 757) that only described one type of management for AGI (Table 4). In the majority of studies, ISR was the main treatment for AGI. Most of the studies had a small number of patients, and they were all conducted in a single centre. The mortality outcome was measured differently in studies depending on the follow-up period. In most studies, mortality was recorded as occurring in a hospital or after thirty days; five studies included in-hospital mortality rates [6] [21] [24] [25] [26], and five studies calculated mortality rates that occurred within a 30-day limit [16] [27] [28] [29] [30]. Common outcomes measured by all studies were mortality, amputation, graft reinfection, and graft related complications. Other important outcomes were not mentioned in most of the studies such as length of stay in hospital, length of stay in ITU, and renal failure.

## 4. Meta-Analysis

Three studies compared surgery with conservative management [21] [22] [23]. Two studies compared ISR with conservative management [21] [23], and Lyons et al. compared EAR with conservative management [22]. Due to variations in research aims between the studies, there were limitations to the morbidity data; consequently, data was only pooled for overall mortality. The overall mortality rate was not significantly different for surgery and conservative management (OR 0.58 [95% CI 0.10-3.38], *P* = 0.55).

**Figure 2:**
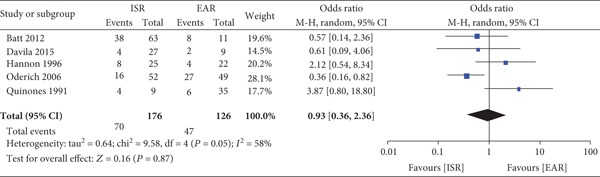
Forrest plot for overall mortality rate between ISR and EAR.

Five studies compared ISR with EAR [13] [17] [18] [19]. There was no significant difference in the overall mortality rate (ISR *n* = 70/176 vs. EAR *n* = 47/126, OR 0.93 [95% CI 0.36-2.36], *P* = 0.87) (Figure 2). The graft occlusion rate was significantly lower in the ISR group vs. the EAR group (*n* = 14/115 vs. *n* = 29/60, OR 0.16 [95% CI 0.07-0.36], *P* < 0.001), and there was no significant heterogeneity between the studies (*P* = 0.43). There was no significant difference in the amputation rate between the surgical treatments (ISR *n* = 9/141 vs. EAR *n* = 8/82, OR 0.75 [95% CI 0.07-8.39], *P* = 0.82).

All types of allografts were pooled together due to the limited number of studies; a separate meta-analysis of the individual types was not possible. Various types of grafts were compared within the ISR group, and the common comparison was made between allograft and prosthetic grafts [12] [14] [31]. There was no significant difference between the groups for the overall mortality rate (allograft *n* = 16/156 vs. prosthetic *n* = 17/122, OR 0.69 [95% CI 0.26-1.85], *P* = 0.46). There was no significant difference in the graft reinfection rate between the groups (allograft *n* = 1/118 vs. prosthetic *n* = 3/88, OR 0.32 [95% CI 0.04-2.52], *P* = 0.28). There was no significant difference in the amputation rate between groups (allograft *n* = 4/118 vs. prosthetic *n* = 2/88, OR 1.14 [95% CI 0.24-5.47], *P* = 0.87). There was no significant difference between the groups in the wound infection rate (allograft *n* = 2/118 vs. prosthetic *n* = 0/88, OR 2.14 [95% CI 0.22-20.93], *P* = 0.51).

## 5. Discussion

### 5.1. Overall Findings

This meta-analysis had 22 studies: 10 cohort studies and 12 comparative studies assessing EAR, ISR, and medical management of AGI. 11 studies were published within the last 10 years, and most of the studies were published within the last 20 years. Research aims were to assess what interventions have the best results for decreasing mortality and morbidity, with a view to create national guidelines in the future. The main finding was that surgery appeared to be the mainstay treatment across all trials for AGI, with poor patient survival when managing patients conservatively. Residual sepsis and patient premorbidity are likely to have a significant role in this; however, this conclusion cannot be drawn from the above data. Our study showed that the in situ graft replacement seemed to be the most popular choice between the various centres closely followed by EAR. A number of different graft types were used for ISR; however, no one graft type is shown to be superior.

It has been believed that the autologous femoral vein is the gold standard. The outcomes of the review do not reveal this as there is not enough data in the established literature. There were only 2 studies that directly compared in situ replacement with allograft and prosthetic graft. Hence, it was not possible to conduct meta-analysis to compare the effects of the two grafts. In one of the studies, the graft occlusion and infection rate were higher with silver-coated prosthetic graft when compared to cryopreserved allograft [12]. However, the allograft use had longer length of stay and lower amputation rate. On the other hand, in the other study [14], the rate of mortality and reoperation rate were higher with the use of prosthetic graft compared to cryopreserved graft.

In this systematic review, due to heterogeneity of the data, only mortality could be compared. It was not possible to compare regional and systemic comorbidity outcomes, as data across all the categories was scanty. However, the ultimate cause of each death could not be extricated from the data. The common outcome, despite intervention, is death, followed by limb amputation and finally graft related complications. The research goals in regard to assessing systemic mortality outcomes (renal failure, myocardial infarctions etc.) were not met as not every study looked at these, making it impossible to compare data due to missing information.

ISR was associated with lower rates of graft occlusion compared to EAR. Previous nonrandomised controlled trials have also observed higher complication rate with EAR [32]. For this reason, a breakdown of both localised and systemic complications and the ultimate outcome for the patients is still needed.

Two important findings from the review support evidence from the previous literature that reported high prevalence of males and bacterial organisms associated with AGI [4] [11]. The risk of AGI was particularly higher in those with blood stream septicaemia and surgical site infection [11]. It is hypothesised that the skin commensal organisms have the potential to infect the graft during its implant. Common organisms found in patients with AGI were staphylococcus species which supports this hypothesis [4].

### 5.2. Strengths

The research material covered in this meta-analysis was comprehensive as it covered research from 27 studies and included 1,365 patients. The inclusion criteria were deliberately broad in order to allow a large amount of research to be included. Research goals took into account a range of interventions including in situ replacement and extra anatomical replacement, and a number of different graft types were taken into consideration [31].

Focusing on the patient journey via longer cohort studies allowed us to have a better idea of prognosis post AGI. AGI has significant consequences in both acute inpatient settings and the long-term health of the patient. Those studies with longer follow-up periods enabled us to better understand outcomes of post AGI. By attempting to look at a broad range of both localised and systemic complications, it was possible to elucidate the gaps in the research in regard to long-term patient outcome. This meta-analysis has revealed that the systemic complications of AGI are especially lacking mention in the research as well as the need for centres to report a wider range of complications in future studies.

Favoured management options were able to be better understood, for example, that surgical intervention is the mainstay treatment. ISR appears to be favoured over EAR, which is a useful point for surgeons encountering their first AGI in the absence of clear guidelines. The choice of graft for ISR needs further research to identify the gold standard.

## 6. Limitations

There was inconsistency in the inclusion criteria of morbidity outcomes in each study. This made it difficult to pool the data as there was a lack of uniformity in the research goals. Every study recorded mortality outcomes; however, the distinction of mortality as a direct link to AGI versus unrelated mortality varied. Some centres recorded any deaths occurring in the same admission but did not include deaths occurring post discharge. There were also limits to follow-up; for example, if patients died within thirty days of AGI treatment, they were classed as a mortality statistic; if they fell out of this remit, they did not. This needs to be addressed in future studies. Consequently, there is both a need to standardise research goals that include equivalent morbidity outcomes and also a need to create a uniform approach to record mortality outcomes, for example, increasing follow-up duration to prevent inaccurate statistics. By standardising research goals across multiple centres in the way, future meta-analyses are likely to hold more weight.

Within previous studies, the comparisons between open and endovascular surgeries was missing and must be included. Factors that were missing from the data included the patient's length of stay as an inpatient, length of stay in ITU, renal failure, and cardiopulmonary complications. As all of the aforementioned factors significantly affect overall health and quality of life, they are essential factors to be included in future research. Importantly, the causes of death were not reported by the studies to show whether they were related to the management of the disease.

There were several limitations to this meta-analysis, including inconsistency between studies in regard to the follow-up time, variable numbers of each cohort group, variable duration of studies, attrition, and potential bias introduced as the authors were often from the same institution. There were also degrees of crossover between cohort groups as some patients were initially managed as medical; however, due to continued infection, they were ultimately managed surgically. It is difficult to compare the results between the different centres as the research goals varied so widely. As our focus was solely on aneurysmal disease, we made our best efforts to exclude studies that included occlusive disease; however, in some studies, this was not clear and might have affected our results due to the retrospective nature of the analysis. In these studies, we made efforts to only analyse the data for aneurysmal disease.

Infected endografts are a more common problem being faced, as a significant proportion of aneurysms are now treated endovascularly [33]. We were unable to perform a subgroup analysis on infected endografts/use of endografting as a method of treating AGI's as data heterogeneity prevented this.

It was noted that there were several areas lacking information across most studies. In particular, the following points were not often included in the literature: length of stay in hospital, length of stay in ITU, open surgery vs. endovascular, and emergency vs. elective surgery. These points are all essential in regard to a better understanding of the complexity of each case and left the data set incomplete. Comparisons between the approaches to the abdominal cavity were not made in any of the studies—i.e. open vs. endovascular surgical options. Furthermore, ideally, one would like to compare autologous allografts and prosthetic grafts; however, we were not able to find enough data to compile it and conduct meta-analysis.

Studies included in this review had certain common biases; for example, in the majority of trials, the authors were often employed by the institutions they collected data from. With the exception of Batt et al. and Quiñones [13] [26], most data was pooled from single centres and as such showed little integration between organisations in regard to management strategies of AGI. Individual preferences of which intervention to choose invariably differed between centres; therefore, the lack of standardisation was bound to have affected the results. The authors' direct clinical/surgical involvement in managing these patients may also have influenced their choice of intervention. Each centre differed in their approach to management, likely due to a lack of consensus on how AGI should be managed. Senior guidance and previous surgical interventions used at each unit were likely to have affected the research options.

As many of the studies were conducted over a long period of time: ranging from <1 year to 7 years, it was inevitable that some subjects dropped out of the trial—this weakened the results via a process of attrition. The longer the duration of study the higher the dropout rate; and as such, a true estimate of patient outcomes cannot be made. As a prospective cohort study is the best strategy for assessing long term prognosis, this is a flaw that is difficult to modify. It is inevitable that a number of patients will choose to unsubscribe; and in respect of their human rights, ethical trials must uphold a patient's wishes.

## 7. Conclusion

AGI is well known to have high mortality and morbidity rates; literature has emphasised this clearly across all studies. There is a need for a multicentre study to be conducted in order to achieve standardised outcomes for patients and set guidelines for best practice. This will ensure a better understanding of how to limit mortality and morbidity. This could then lead to the formulation of “gold standard” guidelines which could be followed by all vascular units across the country, and subsequent data gathered could be monitored via national audits.

These future clinical goals should improve patient outcomes, improve the quality of life post AGI, and limit mortality and morbidity outcomes prospectively.

## Figures and Tables

**Table 1:** Search strategies.

Search terms (keyword and thesaurus subject headings, e.g., MeSH and Emtree)	Database
Aort∗ / AORTA explode /	MEDLINE, EMBASE, CINAHL, PUBMED
Graft∗ / VASCULAR GRAFTING Major/select	MEDLINE, PUBMED
Infect∗ / INFECTION select/explode/major	MEDLINE, EMBASE, CINAHL, PUBMED
Aortic∗ ADJ3 graft∗ ADJ3 infect∗ / “aortic graft infection∗” / Graft ADJ3 infect∗	MEDLINE, EMBASE, CINAHL, PUBMED
SURGICAL WOUND INFECTION explode / GRAFTS major/select	CINAHL
AORTA GRAFT explode	EMBASE

**Table 2:** Most common organisms cultured from infected grafts.

Author (year)	Most common organism/organisms
Ali (2009) [27]	Gram positive species
Batt (2003) [26]	Staphylococcus epidermis
	Streptococcus
	Staphylococcus aureus
Ahmed (2017)	Staphylococcus epidermidis
Chaufour (2017) [16]	Staphylococcus epidermidis
Davilla (2015)	Staphylococcus species
Dimuzio (1996)	Staphylococcus epidermis
	Streptococcus
	Staphylococcus aureus
	Propionibacterium acnes
Dirvin (2015)	Coxiella burnetii
Legout (2011)	Staphylococcus aureus
Murphy (2013)	Polymicrobial
Gabriel (2004) [29]	Staphylococcus coagulase
Harlander-Locke (2014)	Staphylococcus aureus
Hayes (1999) [30]	Streptococcus faecalis
Mirzaie (2006)	Staphylococcus aureus
Bisdas (2011) [12]	Staphylococcus aureus
Quinones (1991)	Staphylococcus epidermidis
Vogt (1998) [14]	Staphylococcus aureus
Belair (1998) [15]	Staphylococcus aureus
Hannon (1996) [18]	Escherichia coli
Lyons (2013) [22]	Escherichia coli
Legout (2011)	Staphylococcus aureus

**Table 3:** Data extracted from the studies analysed: Cohort study results.

Author	Year	No. of patients	Intervention	Average age (yrs)	Sex (male)	Overall mortality	Total length of stay (days)	Amputation	Myocardial infarction	Renal failure	Graft reinfection	Graft related complications	Follow-up (months)
Ali	2009	187	ISR (fem-pop vein)	63.2	63%	30 days-10%, procedure related-14%	21 + ‐8	7.4%	4.3%	12%	—	—	63
Batt	2003	24	ISR (silver-coated graft)	69 (median)	93%	16.6% (peri-op)	—	—	—	—	—	—	17
Ahmed	2017	65	ISR-cryopreserved arterial allograft	65.2	91.5%	16.9% (peri-op)	—	1.4%	4.2%	2.8%	4.2%	18.3%	45
Dimuzio	1996	15	EAR	64	—	13.3%	—	13.3%	—	—	—	13.3%	56
Dirvin	2015	14	ISR (autologous venous reconstruction of the aorta)	69	71%	28% (30 days)	28	0%	—	—	0%	—	—
Gabriel	2004	45	ISR-cryopreserved arterial allograft	61	84%	13% (30 days)	—	8.1%	—	—	—	4.4%	30
Harlander-Locke	2014	220	ISR-cryopreserved arterial allograft	65		9.1%	28	3%			3.6%	7.7%	30
Seeger	2000	36	EAR	61.8	75%	19.4%	—	11.1%	13.9%	11.1%	9%	33.3%	—
Hayes	1999	11	ISR	66 (median)	72.7%	18.2% (30 days)	—	0%	—	9.1%	0%	9.1%	—
Mirzaie	2006	11	Partial removal + ISR with silver-impregnated graft (sartorius flap)	—	72.7%	0%	12 + ‐4	—	—	—	—	—	—

**Table 4:** Data extracted from the studies analysed: comparative study results.

Author	Year	No. of patients	Single centre	No. of treatment groups	Groups (e.g., A, B, and C)	Average age (yrs)	Sex (male)	Overall mortality	Graft occlusion	Graft reinfection	Amputation	Reoperation (graft + operation related)	Total length of stay (days)	Follow-up (months)
Bisdas	2011	33	Y	2	A—ISR-cryopreserved arterial homograft [22] B—ISR-silver-coated grafts [11]	68 (A), 61 (B)	94.1%	13.6% (A) 27.2% (B)	0% (A) 9% (B)	0% (A) 18.2% (B)	4.5% (A) 0% (B)	18.2% (A) 36.3% (B)	Group A: 24 + ‐16 Group B: 16 + ‐12	27 (A) 18 (B)
Hannon	1996	47	Y	2	A—ISR [25] B—EAR [22]	62 (A) 63 (B)	—	32% (A) 18.2% (B)	—	4% (A) 0% (B)	24% (A) 13.6% (B)	24% (A) 0% (B)	80 (A) 56 (B)	—
Oderich	2006	117	Y	2	A—ISR (52) B—EAR (49)	69.4 (A) 66.3 (B)	77%	30.8% (A) 55.1% (B)	11.5% (A) 51% (B)	9.6% (A) 30.6% (B)	0% (A) 12.2% (B)	—	—	—
Pupka	2011	77	Y	3	A—fresh arterial allograft with immunosuppression [24] B—fresh arterial allograft without immunosuppression [26] C—silver-coated prosthesis [27]	57.4 (A) 59.2 (B) 58.4 (C)	96.1%	8.3% (A) 23.1% (B) 11.1% (C)	8.3% (A) 34.6% (B) 7.4% (C)	0% (A) 3.8% (B) 3.7% (C)	0% (A) 11.5% (B) 7.4% (C)	—	—	22.8
Quinones	1991	45	N	2	A—ISR [9] B—EAR (35)		76%	24% 30 day (combined)	33% (combined)	8.8% (combined)	33% (combined)	—	—	35.5
Takano	2014	8	Y	2	A—ISR B—conservative management	66	87.5%	25% (combined)	—	—	—	—	—	—
Vogt	1998	72	Y	2	A—ISR-cryopreserved allograft (38) B—ISR prosthetic graft (34)	61 (A) 63 (A)	—	13.2% (A) 32.4% (B)	—	—	—	7.9% (A) 44.1% (B)	14 (A) 30 (B)	—
Batt	2012	74	N	2	A—ISR (63) B—EAR [11]	69.7 (A) 67.9 (B)	96.3%	60.3% (A: 5 years) 72.7% (B: 5 years)	12.7% (A) 14.8% (B)		4.8% (A) 0% (B)		23 + ‐16 (combined)	41
Belair	1998	23	Y	2	A—percutaneous drain+surgery (ISR)-11 B—direct surgery (ISR)-12	65.9 (A) 68.75 (B)	86.9%	30 days: 0% (A) 50%(B) 1 yr: 9% (A) 66.6% (B)	9.1% (A) 41.6% (B)	36.3% (A) 33.3% (B)	—	27.2% (A) 25.0% (B)	Group A: 40 + ‐17 Group B: 25 + ‐15	24 (A) 48 (B)
Lyons	2013	13	Y	2	A—EAR [9] B—conservative [4]	—	72.7%	55.6% (A) 100% (B)		—	—	—	—	29
Davila	2015	36	Y	2	A—ISR [27] B—EAR [9]	69 (combined)	83.3%	14.8% (A) 22.2% (B)	—	—	—	—	—	47.5
Legout	2011	54	Y	2	A—ISR (45) B—conservative [9]	—	—	22.2% (A) 22.2% (B)	—	—	—	—	—	12

## Abbreviations

AAA: Aortic abdominal aneurysm
AGI: Aortic graft infections
EAR: Extra-anatomical repair
EVAR: Endovascular aortic repair
ISR: In situ graft replacement.

## Additional Points

*Key Findings*. In situ replacement was compared with extra-anatomical replacement. There was no significant difference in the mortality rate or amputation rate. The graft occlusion rate was significantly lower in ISR (*P* < 0.001). Allografts and prosthetic grafts were compared, and no significant difference in mortality and graft/wound infection rates was identified.

*Take Home Message*. There is a need for a multicentre study to be conducted in order to achieve standardised outcomes for patients and set guidelines for best practice. This will ensure a better understanding of how to limit mortality and morbidity and can lead to formulation of “gold standard” guidelines.
